# Effects of dance therapy in women with breast cancer: A systematic review protocol

**DOI:** 10.1371/journal.pone.0257948

**Published:** 2022-06-24

**Authors:** Natália Silva da Costa, Amanda Suzane Alves da Silva, João Simão de Melo-Neto

**Affiliations:** Federal University of Pará (UFPA), Belém, Pará, Brazil; Universidade Federal da Bahia, BRAZIL

## Abstract

**Background:**

Cancer is an important public health problem with an increasing global incidence in the recent decades. Breast cancer has become the leading cause of death in women worldwide. Women suffering from breast cancer, as well as survivors, may experience some adverse effects of treatment–including cancer-related fatigue, sleep disorders, and pain–which may manifest alone or in combination with other symptoms. Non-pharmacological interventions, such as physical activity, have been associated with improvements in these adverse effects. This study aims to evaluate the effects of dance therapy in women with breast cancer.

**Methods:**

We will perform a systematic review according to the Cochrane methodology. An overall search strategy will be developed and adapted for PubMed, Virtual Health Library, PEDro, SciELO, SciVerse Scopus, Cochrane Library, and Web of Science using the descriptors “Dance therapy” or “Dancing” and “Breast neoplasms” or “Breast cancer.” The size of the intervention effect (Z) will be calculated for each outcome included in this review. Outcomes will be pain, cancer-related fatigue, sleep disturbance, body image and depression in women with breast cancer. Quality assessment will be performed using the Cochrane instrument. Metanalysis, if plausible, will be performed using Review Manager 5.3.

**Discussion:**

Studies have reported positive results of dance therapy as a non-pharmacological intervention in women with breast cancer. Thus, it is expected that robust and conclusive evidence of the effects of dance therapy during or after treatment (radiotherapy, chemotherapy, hormone therapy, and/or surgery) can be obtained.

**Trial registration:**

Systematic review registration: CRD42020152876. (S1 File).

## Introduction

Cancer has become a public health problem that has been growing proportionally among chronic diseases. Currently, it is the second leading cause of death globally. Its incidence has increased in both developed and developing countries, likely due to increasing exposure to risk factors and prolonged life expectancy [1]. Breast cancer has become a primary health issue in our society as a result of an aging population, emerging alongside other chronic, non-communicable diseases [2]. In Brazil, breast cancer is the most prevalent type of cancer and has the worst prognosis in women. Women with breast cancer face a high risk of developing metabolic diseases, and chronic pain [3].

Adverse effects of the neoplasm itself, or of the treatment, are predominantly symptoms such as pain, cancer-related fatigue, and sleep disorders that manifest themselves alone or in combination with other symptoms [4]. One of the most commonly reported symptoms is fatigue related to cancer (having multifactorial pathophysiology) and oncologic pain (triggered by the effects of endogenous opioids) [5, 6]. These symptoms reduce an individual’s activities of daily life, contributing to muscle protein degradation without a corresponding increase in protein synthesis [7]. Women tend to have high degrees of negative body image perceptions, which can directly affect their self-esteem and stress levels. This can trigger an imbalance in their mental health [8], which can result in depression. Cancer patients can have symptoms and adverse effects that persist even after the completion of treatment [5, 6]. Thus, it is important to identify interventions that can reduce symptoms such as pain, fatigue, sleep disturbance, negative body image, and depression.

Oncology offers a variety of features designed to help patients cope with the adverse effects of treatment. Patients have access to medications and therapeutic interventions performed by multidisciplinary teams [9]. Non-medicinal interventions have been suggested to complement these, to further minimize adverse effects. In this scenario, physical activity was practiced. Physically active individuals show a reduction in the signs and symptoms of the disease as well as adverse effects of the drugs [10]. Currently, there is an increasing number of studies [11–13] evaluating the effects of dance therapy in patients with breast cancer.

Dance therapy is a modality of physical exercise that uses movement as a psychotherapeutic tool to promote integration, based on the assumption of interconnection between an individual’s body and mind [14]. When performed with music, this can become especially motivational [15]. In this way, dance therapy as a modality of physical exercise can have physiological benefits that improve physical and mental health.

Regarding its physical aspects, exercise may favor pain reduction through muscle toning and noradrenergic system activation that occurs with the release of catecholamines. This can modulate the nociceptive pathway, which may cause an increase in pain thresholds [16]. In addition, regular physical exercise can reduce oncologic fatigue and improve sleep quality by regulating pro- and anti-inflammatory cytokines [17].

The mental aspect of dance therapy utilizes the expressiveness of an individual’s spontaneous movements, based on the assumption of interconnection of body and mind. This can allow the externalization of their feelings at that moment in their life, some of which can trigger the development of depression [18]. Thus, one of the mechanisms of physical exercise is an increase in neurotransmitter substances that influence hippocampal neurogenesis, promoting the release of beta-endorphins, vascular endothelial growth factor, brain-derived neurotrophic factor, and serotonin. These substances are directly related to well-being and can favor positive perceptions of self-image among women [19].

With the understanding of these effects of dance therapy, we asked the question "Can dance therapy as a modality of physical exercise reduce symptoms in women with breast cancer?” Thus, this study aims to evaluate the effects of dance therapy on pain, cancer-related fatigue, sleep disturbance, body image and depression in women with breast cancer. In this sense, our hypothesis is to check the effects of dance therapy as a motion therapy option to reduce symptoms in women with breast cancer.

## Methods/Design

### Type of study

A systematic review of literature with a meta-analysis (if possible) of intervention trials, in accordance with the recommendations of the Preferred Report Items for Systematic Reviews and Meta-analyses (PRISMA) statement [20] (S2 File).

### Eligibility criteria

#### Inclusion criteria

Controlled clinical trials and pilot studies, both randomized or non-randomized, in women with breast cancer (stages I to III), regardless of the age group that participated in dance therapy, performed throughout the treatment (radiotherapy, chemotherapy, hormone therapy, and/or surgery—isolated or combined), being the presence in all sessions for participants. There will be no restriction with respect to the date of search for the studies, and all possible evidence will be analyzed.

#### Exclusion criteria

No studies other than randomized clinical trials or pilot studies, either randomized or non-randomized, will be included.

### Outcomes

The effects of dance therapy on pain, cancer-related fatigue, sleep disturbance, body image, and depression in women with breast cancer.

### Research strategies

A search strategy will be created and adapted for searching the databases Pubmed, Virtual Health Library, PEDro, SciELO, SciVerse Scopus, Cochrane Library, and Web of Science to identify studies involving the above-mentioned interventions. RCT registry sites will be consulted for ongoing studies on the topic (Rebec, Clinicaltrial.gov). Articles written in Portuguese, English, and/or Spanish in accordance with the PICOS protocol (population, intervention, comparison, outcome, and type of study) will be considered. MeSH terms used for our searches are “Dance therapy” or “Dancing” and “Breast neoplasms” or “Breast cancer.”

### Data collection and analysis

#### Selection of studies

**Fig 1:**
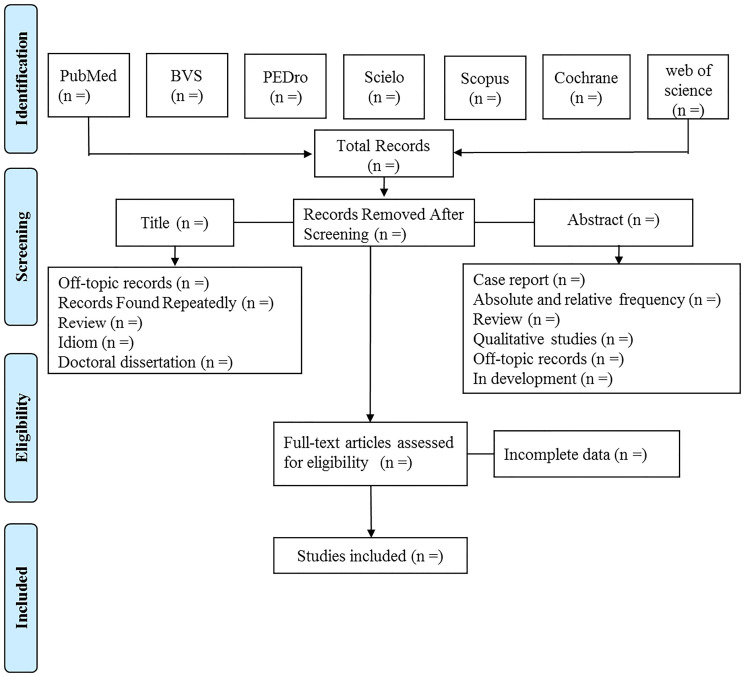
Study selection for review.

For each search strategy, two reviewers will independently evaluate the studies retrieved from the databases in the following order: title, abstract, and full reading (Fig 1). All studies potentially eligible for inclusion in the review will be selected for full reading. In case of disagreement, a third reviewer (JSMN) will be consulted.

#### Data extraction and management

Data extraction for eligible studies will be performed by two reviewers (NSC and ASAS) who will independently extract data from articles that meet the inclusion criteria. All data will be tabulated in an Excel spreadsheet and Review Manager software made by the researchers.

A standardized form will be used to extract the following information: study characteristics (design), participants (type of cancer and treatment, mean age), interventions (dance therapy and protocol details), and clinical outcomes (pain, cancer-related fatigue, sleep disturbance, body image, and depression).

Studies that do not fully meet the inclusion criteria will be excluded and tabulated with their justification for exclusion (Table 1). The characteristics of the selected studies are listed in Table 2.

**Table 1:** Study characteristics related to the number of participants, inclusion and exclusion criteria.

Author / Year	No. of participants	Inclusion criteria	Exclusion criteria
			
			
			
			

**Table 2:** Study characteristics related to the configuration: Title, authors, years; type of cancer; number of participant per group; mean of age; characterization of dance therapy (protocol details); outcome; follow-up (simplified); results; score the PEDro scale.

Title /Author/Year	Type of Cancer	No. of participant per group	Mean of Age	Characteri-zation of Dance Therapy	Outcome	Follow-up	Results	PEDro
								
								
								

#### Evidence quality and risk of bias

The evidence quality and risk of bias in the studies selected for this systematic review will be assessed using the PEDro scale [21, 22]. The PEDro scale is used in the analysis of studies of physical therapy. This scale assesses the following domains: allocation generation, concealment of allocation, masking (of participants and researchers), presence of incomplete data, reporting bias of information, and other types of biases. The answers to these domains may be “Yes,” “No” or “Where” [21].

#### Statistical analysis

The size of the intervention effect (Z) will be calculated for each study included in this review using mean difference (DM) or standardized DM as measures of effect. The effects models (fixed or random) will be used to estimate the effect of treatment in women. The study models used in the analysis will be defined according to the similarity of the effects of interest among the studies. Fixed effect models will be applied to similar studies, while random effects model will be applied to the others. The absence of bias in relation to estimates against sample size will be analyzed using a funnel plot. The degree of heterogeneity of dates will be evaluated according to I^2^ following the recommendations of the Cochrane Manual for Systematic Reviews of Interventions version 6.1, 2020 [23]. Statistical significance will be set at P <0.05. Review Manager software will be used for all analyses, including meta-analysis if possible [24].

## Discussion

Literature has suggested that dance therapy performed during oncologic treatment, under intensive supervised guidance, appears to be effective in reducing treatment side effects in women with breast cancer. However, the results of this non-medical therapy are not clear, and it is difficult to systematically analyze the dance therapy programs for women with breast cancer.

In existing literature, there are studies with clinical trials (both randomized and non-randomized) and pilot studies (both randomized and non-randomized). These research designs were chosen because they allow us to evaluate the best available evidence. The present systematic review (and meta-analysis, if possible) aims to demonstrate the significant effects of dance therapy as a treatment for women with breast cancer.

Specifically, the effects of this therapy on pain, cancer-related fatigue, sleep disturbance, body image and depression will be analyzed. Thus, we can obtain robust and conclusive evidence regarding the effects of dance therapy to support clinical practice, in addition to contributing high-quality studies on the subject.

## Limitations

This systematic review with meta-analysis may be limited if there is a scarcity of appropriate studies in literature, methodological quality of the studies, heterogeneity of experimental protocols, and absence of data from studies for analysis.

## Supporting information

S1 File PROSPERO international prospective register of systematic reviews. (PDF)

S2 File PRISMA-P (Preferred Reporting Items for Systematic review and Meta-Analysis Protocols) 2015 checklist: Recommended items to address in a systematic review protocol. (PDF)
