# Supplementary material for: Effects of dance therapy in women with breast cancer: A systematic review protocol
Source: PLoS One. 2022 Jun 24;17(6):e0257948. doi: 10.1371/journal.pone.0257948 (PMC9231811; doi:10.1371/journal.pone.0257948)
Supplement: S1 File — (PDF) [file pone.0257948.s001.pdf]

NHS
National Institute for Health Research

UNIVERSITY of York
Centre for Reviews and Dissemination

Systematic review

1. * Review title.

Give the title of the review in English

Effects of therapeutic dance in women with breast cancer: a systematic review

2. Original language title.

For reviews in languages other than English, give the title in the original language. This will be displayed with the English language title.

3. * Anticipated or actual start date.

Give the date the systematic review started or is expected to start.

29/07/2019

4. * Anticipated completion date.

Give the date by which the review is expected to be completed.

31/12/2020

5. * Stage of review at time of this submission.

Tick the boxes to show which review tasks have been started and which have been completed. Update this field each time any amendments are made to a published record.

Reviews that have started data extraction (at the time of initial submission) are not eligible for inclusion in PROSPERO. If there is later evidence that incorrect status and/or completion date has been supplied, the published PROSPERO record will be marked as retracted.

This field uses answers to initial screening questions. It cannot be edited until after registration.

The review has not yet started: No

Review stage	Started	Completed
Preliminary searches	Yes	Yes
Piloting of the study selection process	Yes	Yes
Formal screening of search results against eligibility criteria	Yes	Yes
Data extraction	Yes	Yes
Risk of bias (quality) assessment	Yes	Yes
Data analysis	Yes	No

Provide any other relevant information about the stage of the review here.

6. * Named contact.

The named contact is the guarantor for the accuracy of the information in the register record. This may be any member of the review team.

Natália Silva da Costa

Email salutation (e.g. "Dr Smith" or "Joanne") for correspondence:

Miss Silva da Costa

7. * Named contact email.

Give the electronic email address of the named contact.

ns.costa5@gmail.com

8. Named contact address

Give the full institutional/organisational postal address for the named contact.

Street Angélicas, 02 Health sector: Parque Verde \ Belém - PA, 66635745

9. Named contact phone number.

Give the telephone number for the named contact, including international dialling code.

(55)91981925560

10. * Organisational affiliation of the review.

Full title of the organisational affiliations for this review and website address if available. This field may be completed as 'None' if the review is not affiliated to any organisation.

Federal University of Pará (UFPA)

Organisation web address:

11. * Review team members and their organisational affiliations.

Give the personal details and the organisational affiliations of each member of the review team. Affiliation refers to groups or organisations to which review team members belong. **NOTE: email and country now MUST be entered for each person, unless you are amending a published record.**

Miss Natalia Silva da Costa. Federal University of Pará (UFPA), Belém, PA, Brazil. Clinical and Experimental Research Unit of the urogenital system
Miss Thalita da Luz Costa. Federal University of Pará (UPA), Belém, PA, Brazil. Clinical and Experimental Research Unit of the urogenital system
Dr João Simão de Melo-Neto. Federal University of Pará (UFPA), Belém, PA, Brazil. Clinical and Experimental Research Unit of the urogenital system
Miss Amanda Suzane Alves da Silva. Universidade Federal do Pará

12. * Funding sources/sponsors.

Details of the individuals, organizations, groups, companies or other legal entities who have funded or sponsored the review.

no

Grant number(s)

State the funder, grant or award number and the date of award

13. * Conflicts of interest.

List actual or perceived conflicts of interest (financial or academic).

None

14. Collaborators.

Give the name and affiliation of any individuals or organisations who are working on the review but who are not listed as review team members. **NOTE: email and country must be completed for each person, unless you are amending a published record.**

Miss Natália Silva da Costa. Federal University of Pará (UFPA), Belém, PA, Brazil.
Mr Dionata Barbosa da Costa. Federal University of Pará (UFPA), Belém, PA, Brazil.
Mr Fabiana de Campos Gomes. School of Medicine of São José do Rio Preto (FAMERP), São José do Rio Preto, SP, Brazil.
Miss Thalita da Luz Costa. Federal University of Pará (UPA), Belém, PA, Brazil. Clinical and Experimental Research Unit of the urogenital system
Dr João Simão de Melo-Neto. Federal University of Pará (UFPA), Belém, PA, Brazil.

15. * Review question.

State the review question(s) clearly and precisely. It may be appropriate to break very broad questions down into a series of related more specific questions. Questions may be framed or refined using PI(E)COS or similar where relevant.

What are the effects of dance therapy on muheres with breast cancer?

16. * Searches.

State the sources that will be searched (e.g. Medline). Give the search dates, and any restrictions (e.g. language or publication date). Do NOT enter the full search strategy (it may be provided as a link or attachment below.)

The research will be carried out according to the PRISMA model (report items for systematic analysis and meta-analysis).

Database: PubMed, VHL, SciELO, Scopus, Cochrane BIREME, Web of Science and PEDro

The search terms will be: ((("Dancing"[MeSH]) OR ("Dance Therapy"[MeSH])) AND ("Breast

Neoplasms"[MeSH] or "breast cancer")

Inclusion requirements are defined as:

• Articles published in English, Portuguese or Spanish

• Involve the descriptors listed in the variables

• Non-randomized randomized clinical trial

• Published in journals indexed to Qualis and / or impact factors.

Excluded studies are:

• Non-quantitative

• Non-primary research

• Different dance intervention

• Those published in other languages

• Systematic reviews, dissertations or theses, case studies

17. URL to search strategy.

Upload a file with your search strategy, or an example of a search strategy for a specific database, (including the keywords) in pdf or word format. In doing so you are consenting to the file being made publicly accessible. Or provide a URL or link to the strategy. Do NOT provide links to your search **results**.

Alternatively, upload your search strategy to CRD in pdf format. Please note that by doing so you are consenting to the file being made publicly accessible.

Do not make this file publicly available until the review is complete

18. * Condition or domain being studied.

Give a short description of the disease, condition or healthcare domain being studied in your systematic review.

O câncer de mama tornou-se um problema de saúde primário em nossa sociedade como resultado do envelhecimento populacional, emergindo ao lado de outras doenças crônicas não transmissíveis. No Brasil, o câncer de mama é a forma mais prevalente de câncer com pior prognóstico em mulheres. Pacientes com câncer de mama e sobreviventes têm maior risco de desenvolver disfunções metabólicas e dor crônica. Além disso, esses possuem altos graus de percepção negativa em relação à imagem corporal, o que afeta diretamente sua autoestima e níveis de estresse e leva ao desequilíbrio da saúde mental, o que pode resultar em depressão. Em relação aos efeitos principais do tratamento, observado-se que fadiga, distúrbios do sono e dor podem se manifestar isoladamente ou combinados que podem persistir mesmo após o término do tratamento. Um dos sintomas mais comumente relatado é a fadiga relacionada ao câncer de fisiopatologia multifatorial e a dor desencadeada pelos efeitos dos opióides endógenos, ambas impactam na redução das atividades de vida diária do indivíduo o que pode contribuir para degradação da proteína muscular sem um aumento correspondente da síntese proteica.

Pacientes com câncer apresentam [illegible] efeitos adversos [illegible] sintomas.

Dessa forma, o exercício físico estimula o metabolismo oxidativo do músculo esquelético, favorecendo o aumento nos níveis sistêmicos de Interleucina-6, enquanto os níveis de fator de necrose tumoral alfa diminuem. além de favorecer o tônus muscular o ocorre a ativação sistema noradrenérgico com a liberação de catecolaminas modulam a via nociceptiva podendo provocar uma elevação dos limiares de dor.

19. * Participants/population.

Specify the participants or populations being studied in the review. The preferred format includes details of both inclusion and exclusion criteria.

Women with breast cancer, who participated in a dance therapy program during the period of cancer treatment (surgery, chemotherapy, radiotherapy, hormone therapy), aged over 18 years, regardless of the type of cancer.

20. * Intervention(s), exposure(s).

Give full and clear descriptions or definitions of the interventions or the exposures to be reviewed. The preferred format includes details of both inclusion and exclusion criteria.

Based on the literature, studies using dance therapy will be included as a contributing factor in reducing symptoms related to breast cancer treatment in women undergoing treatment (surgery, chemotherapy, radiotherapy, hormone therapy).

21. * Comparator(s)/control.

Where relevant, give details of the alternatives against which the intervention/exposure will be compared (e.g. another intervention or a non-exposed control group). The preferred format includes details of both inclusion and exclusion criteria.

Only studies that performed dance therapy and performed comparison with experimental or control group were considered.

22. * Types of study to be included.

Give details of the study designs (e.g. RCT) that are eligible for inclusion in the review. The preferred format includes both inclusion and exclusion criteria. If there are no restrictions on the types of study, this should be stated.

During a systematic review, the following databases were used: PubMed, VHL, SciELO, Scopus, Cochrane BIREME, Web of Science and PEDro. The [illegible] studies conducted [illegible] performed by PRISMA. Articles written in Portuguese, English or Spanish [illegible] exclusion criteria according to the [illegible] framework (population, intervention, comparison, outcome and type of study).

The study design was aimed at developing an objective for a bibliographic search of evidence and presentation of intervention through dance therapy for women with breast cancr. The descriptors chosen for this screening were (("Dancing" [Mesh]) OR ("Dance Therapy" [Mesh])) AND ("Breast Neoplasms" [Mesh] or "breast cancer")

23. Context.

Give summary details of the setting or other relevant characteristics, which help define the inclusion or exclusion criteria.

The therapy through dance presents as characteristic the expressiveness of the spontaneous movement of the individual, based on the assumption of the interconnection of the body and mind, enabling the externalization of the feelings that most guide this moment of the patient's life of which some of them may be triggers for the development of depression, thus the increase of neurotransmitter substances influencing a hippocampal neurogenesis promoting the release of beta-endorphins , vascular endothelial growth factor (VEGF), brain-derived neurotrophic factor (BDNF) and serotonin that are related to well-being, and may favor the positive perception of women in relation to self-image.

Cancer patients have symptoms and adverse effects that may remain even after the end of treatment. One of the most commonly reported symptoms is multifactorial pathophysiology cancer-related fatigue and pain triggered by the effects of endogenous opioids, both impact on the reduction of the individual's activities of daily living, which can contribute to muscle protein degradation without a corresponding increase in protein synthesis.

Thus, physical exercise stimulates the oxidative metabolism of skeletal muscle, favoring the increase in systemic levels of Interleukin-6, while levels of tumor necrosis factor alpha decrease. in addition to favoring muscle tone, the activation of the noradrenergic system occurs with the release of catecholamines modulate the nociceptive pathway and may cause an elevation of pain thresholds.

24. * Main outcome(s).

Give the pre-specified main (most important) outcomes of the review, including details of how the outcome is defined and measured and when these measurement are made, if these are part of the review inclusion criteria.

The main symptoms present in the studies were fatigue, pain, sleep disorder, depression and body image

* Measures of effect

Please specify the effect measure(s) for you main outcome(s) e.g. relative risks, odds ratios, risk difference, and/or 'number needed to treat.

- Body Image Scale
- Facit-Fatigue Scale
- Beck Depression Inventory
- Brief pain inventory
- Sleep quality index in Pittsburgh

25. * Additional outcome(s).

List the pre-specified additional outcomes of the review, with a similar level of detail to that required for main outcomes. Where there are no additional outcomes please state ‘None’ or ‘Not applicable’ as appropriate

to the review

Physical and psychosocial variables

* Measures of effect

Please specify the effect measure(s) for you additional outcome(s) e.g. relative risks, odds ratios, risk difference, and/or 'number needed to treat.

Results regarding the study population were presented as descriptive. The size of the intervention effect was calculated for each study included in this review by using the mean difference (MD) or standardized mean difference. When there was low heterogeneity in the data, a meta-analysis of fixed effects was performed to estimate the effect of the treatment on the patients. If there was high heterogeneity, we used a model for random effects. The absence of publication bias was analyzed using a funnel plot. The probability of a type I error in the statistical tests was evaluated for p0.05. All data analyses were conducted using Review Manager (version 5.3; the Cochrane Collaboration, London, United Kingdom).

26. * Data extraction (selection and coding).

Describe how studies will be selected for inclusion. State what data will be extracted or obtained. State how this will be done and recorded.

During the systematic review, three databases: PubMed, VHL, SciELO, Scopus, Cochrane BIREME, Web of Science and PEDro. The inclusion and exclusion criteria were performed by PRISMA. The first extraction was by tithing, followed by the rule and finally the reading of the article in the integrated, for a final analysis. The articles with Disagreements were discussed among the members of the research, to reach a consensus. This systemic review included studies that adhered to the PICOS criteria.

27. * Risk of bias (quality) assessment.

State which characteristics of the studies will be assessed and/or any formal risk of bias/quality assessment tools that will be used.

The quality of the methodology used in the articles selected in this systematic review was analyzed using the PEDro scale. Each study will be evaluated by two independent researchers and their results will be compared to a final consensus on the risk of bias. RevMan 5.3 will be used.

28. * Strategy for data synthesis.

Describe the methods you plan to use to synthesise data. This **must not be generic text** but should be **specific to your review** and describe how the proposed approach will be applied to your data. If meta-analysis is planned, describe the models to be used, methods to explore statistical heterogeneity, and software package to be used.

With the synthesis of the main evidencefound, the types of dance used, impacts of dance on the main

variables of each study, number of sessions, days a week, class time, ensuring that there is a consensus between the items between the use of dance to improve certain physical variables, and its use in improving psychosocial variables in women undergoing post-breast cancer treatment.

29. * Analysis of subgroups or subsets.

State any planned investigation of 'subgroups'. Be clear and specific about which type of study or participant will be included in each group or covariate investigated. State the planned analytic approach.

no

30. * Type and method of review.

Select the type of review, review method and health area from the lists below.

Type of review

Cost effectiveness
No

Diagnostic
No

Epidemiologic
No

Individual patient data (IPD) meta-analysis
No

Intervention
No

Meta-analysis
No

Methodology
No

Narrative synthesis
No

Network meta-analysis
No

Pre-clinical
No

Prevention
No

Prognostic
No

Prospective meta-analysis (PMA)
No

Review of reviews
No

Service delivery
No

Synthesis of qualitative studies
No

Systematic review
No

Other
No

Health area of the review

Alcohol/substance misuse/abuse
No

Blood and immune system
No

Cancer
No

Cardiovascular
No

Care of the elderly
No

Child health
No

Complementary therapies
No

COVID-19
No

Crime and justice
No

Dental
No

Digestive system
No

Ear, nose and throat
No

Education
No

Endocrine and metabolic disorders
No

Eye disorders
No

General interest
No

Genetics
No

Health inequalities/health equity

No

Infections and infestations
No

International development
No

Mental health and behavioural conditions
No

Musculoskeletal
No

Neurological
No

Nursing
No

Obstetrics and gynaecology
No

Oral health
No

Palliative care
No

Perioperative care
No

Physiotherapy
No

Pregnancy and childbirth
No

Public health (including social determinants of health)
No

Rehabilitation
No

Respiratory disorders
No

Service delivery
No

Skin disorders
No

Social care
No

Surgery
No

Tropical Medicine
No

Urological
No

Wounds, injuries and accidents
No

Violence and abuse
No

31. Language.

Select each language individually to add it to the list below, use the bin icon to remove any added in error.
Inglês

There is an English language summary.

32. * Country.

Select the country in which the review is being carried out. For multi-national collaborations select all the countries involved.

Brazil

33. Other registration details.

Name any other organisation where the systematic review title or protocol is registered (e.g. Campbell, or The Joanna Briggs Institute) together with any unique identification number assigned by them. If extracted data will be stored and made available through a repository such as the Systematic Review Data Repository (SRDR), details and a link should be included here. If none, leave blank.

34. Reference and/or URL for published protocol.

If the protocol for this review is published provide details (authors, title and journal details, preferably in Vancouver format)

Add web link to the published protocol.

Or, upload your published protocol here in pdf format. Note that the upload will be publicly accessible.

No I do not make this file publicly available until the review is complete

Please note that the information required in the PROSPERO registration form must be completed in full even if access to a protocol is given.

35. Dissemination plans.

Do you intend to publish the review on completion?

Yes

Give brief details of plans for communicating review findings.?

36. Keywords.

Give words or phrases that best describe the review. Separate keywords with a semicolon or new line. Keywords help PROSPERO users find your review (keywords do not appear in the public record but are included in searches). Be as specific and precise as possible. Avoid acronyms and abbreviations unless these are in wide use.

Dancing OR Dance Therapy AND Breast Neoplasms OR breast cancer

37. Details of any existing review of the same topic by the same authors.

If you are registering an update of an existing review give details of the earlier versions and include a full bibliographic reference, if available.

38. * Current review status.

Update review status when the review is completed and when it is published.New registrations must be ongoing so this field is not editable for initial submission.
Please provide anticipated publication date

Review_Ongoing

39. Any additional information.

Provide any other information relevant to the registration of this review.

40. Details of final report/publication(s) or preprints if available.

Leave empty until publication details are available OR you have a link to a preprint (NOTE: this field is not editable for initial submission). List authors, title and journal details preferably in Vancouver format.

Give the link to the published review or preprint.
